# Sensory Percepts Elicited by Chronic Macro-Sieve Electrode Stimulation of the Rat Sciatic Nerve

**DOI:** 10.3389/fnins.2021.758427

**Published:** 2021-10-07

**Authors:** Nikhil S. Chandra, Weston M. McCarron, Ying Yan, Luis C. Ruiz, Eric G. Sallinger, Nathan K. Birenbaum, Harold Burton, Leonard Green, Daniel W. Moran, Wilson Z. Ray, Matthew R. MacEwan

**Affiliations:** ^1^Department of Biomedical Engineering, Washington University in St. Louis, St. Louis, MO, United States; ^2^Department of Neurosurgery, Washington University School of Medicine in St. Louis, St. Louis, MO, United States; ^3^Department of Biology, Washington University in St. Louis, St. Louis, MO, United States; ^4^Department of Neuroscience, Washington University School of Medicine in St. Louis, St. Louis, MO, United States; ^5^Department of Psychological & Brain Sciences, Washington University in St. Louis, St. Louis, MO, United States

**Keywords:** peripheral nerve stimulation, macro-sieve electrode, regenerative electrode, sensory feedback, sensorimotor restoration, nerve regeneration, rat behavior, sciatic nerve

## Abstract

**Objective:** Intuitive control of conventional prostheses is hampered by their inability to provide the real-time tactile and proprioceptive feedback of natural sensory pathways. The macro-sieve electrode (MSE) is a candidate interface to amputees’ truncated peripheral nerves for introducing sensory feedback from external sensors to facilitate prosthetic control. Its unique geometry enables selective control of the complete nerve cross-section by current steering. Unlike previously studied interfaces that target intact nerve, the MSE’s implantation requires transection and subsequent regeneration of the target nerve. Therefore, a key determinant of the MSE’s suitability for this task is whether it can elicit sensory percepts at low current levels in the face of altered morphology and caliber distribution inherent to axon regeneration. The present *in vivo* study describes a combined rat sciatic nerve and behavioral model developed to answer this question.

**Approach:** Rats learned a go/no-go detection task using auditory stimuli and then underwent surgery to implant the MSE in the sciatic nerve. After healing, they were trained with monopolar electrical stimuli with one multi-channel and eight single-channel stimulus configurations. Psychometric curves derived by the method of constant stimuli (MCS) were used to calculate 50% detection thresholds and associated psychometric slopes. Thresholds and slopes were calculated at two time points 3 weeks apart.

**Main Results:** For the multi-channel stimulus configuration, the average current required for stimulus detection was 19.37 μA (3.87 nC) per channel. Single-channel thresholds for leads located near the nerve’s center were, on average, half those of leads located near the periphery (54.92 μA vs. 110.71 μA, or 10.98 nC vs. 22.14 nC). Longitudinally, 3 of 5 leads’ thresholds decreased or remained stable over the 3-week span. The remaining two leads’ thresholds increased by 70–74%, possibly due to scarring or device failure.

**Significance:** This work represents an important first step in establishing the MSE’s viability as a sensory feedback interface. It further lays the groundwork for future experiments that will extend this model to the study of other devices, stimulus parameters, and task paradigms.

## Introduction

Conventional prostheses’ lack of tactile and proprioceptive feedback is one of several factors contributing to their abandonment. Amputees compensate for this deficiency by devoting excessive visual attention for the effective control of their devices. Contemporary neuroprosthetics research aims to integrate sensory feedback with prosthetic technologies (Biddiss and Chau, 2007). Truncated peripheral nerves are ideal targets for sensorimotor intervention due to their retained ability to transmit motor and sensory signals associated with their original innervation targets (Dhillon et al., 2004), coupled with an axonal somatotopy that extends to the spinal cord (Hallin, 1990; Brushart, 1991). Implanted electrodes interfaced with residual nerve tissue can relay information from prosthetic sensors, providing a means to reintroduce sensory feedback to the central nervous system (e.g., Dhillon et al., 2004; Raspopovic et al., 2014; Tan et al., 2015; Davis et al., 2016).

An electrode’s ability to selectively recruit somatotopically organized axon clusters determines the extent to which elicited sensations are perceived at distinct locations in the phantom limb. Axons closer to an electrode’s metallized leads require lower activating currents, which are undamaging to nerve tissue and associated with realistic percepts without paresthesia. The extraneural cuff electrode’s (ECE’s) placement of leads at the nerve perimeter confers limited control over interior axons (Veraart et al., 1993). Various alternatives offering progressively greater intimacy with and selective control over target axons have emerged. The flat interface nerve electrode (FINE) brings interior axons closer to the periphery and within the range of surface leads by flattening the nerve (Tan et al., 2014, 2015; Charkhkar et al., 2018). The longitudinal intrafascicular electrode (LIFE) is a thin, insulated filament that is threaded axially through the nerve so that its exposed tip lies at the center of a nerve fascicle. Control of multiple fascicles requires multiple filaments, making this electrode an impractical choice for applications requiring selective control of the entire nerve (Lefurge et al., 1991; Dhillon et al., 2004). The transverse intrafascicular multichannel electrode (TIME) is of similar design but penetrates the nerve perpendicularly. This allows leads distributed along its length to interface separate fascicles, enabling selective control of a wider area of nerve (Boretius et al., 2010; Raspopovic et al., 2014; Petrini et al., 2019). The Utah slanted electrode array (USEA) provides wide coverage and high selectivity by penetrating the nerve with 96 metallic tines arranged in a 2-dimensional grid. The tines penetrate to varying depths so that an axon cluster anywhere within the nerve falls under the ambit of a nearby tine (Davis et al., 2016).

Another interface class with potential sensory feedback applications is the regenerative electrode (RE). The archetypal RE is a flat disk perforated with holes called “transit zones.” During implantation, the RE is secured between the stumps of a transected nerve using attached silicone conduits. Guided by these conduits, proximal stump axons regenerate through the transit zones and create a robust mechanical coupling between the RE and nerve structure. Interspersed leads enable selective recording and stimulation of axons across the nerve.

Early RE designs were wafers of Teflon (Marks, 1969) or epoxy (Mannard et al., 1974) with a limited number of drilled transit zones. The emergence of microfabrication technologies for silicon (Edell, 1986; Akin et al., 1994), and later the more biocompatible polyimide (Navarro et al., 1998; Stieglitz et al., 2000), brought with it the prospect of creating high transparency devices with transit zones small enough to interface axons at the individual level. Although axon regeneration has been reported through holes as small as 2 μm (Bradley et al., 1992), such small diameters cause constrictive axonopathy and are obstructive to axon growth at the levels required for sensorimotor restoration. Thus, conventional RE design has long been a compromise between keeping transit zone diameters small enough to interface with as few axons as possible and not inhibiting regeneration altogether; an ideal diameter of between 40 and 65 μm has been suggested previously (Navarro et al., 1996). Efforts to increase the number of transiting axons–and hence functional recovery–have typically focused on increasing the number of transit zones (Wallman et al., 2001; Lago et al., 2007). However, reports that the number of myelinated fibers distal to the RE eventually reach control values (Ceballos et al., 2002) are more reflective of the branching inherent to axon regeneration than the nominal increase in axons traversing the RE’s plane of activation (Negredo et al., 2004).

**FIGURE 1:**
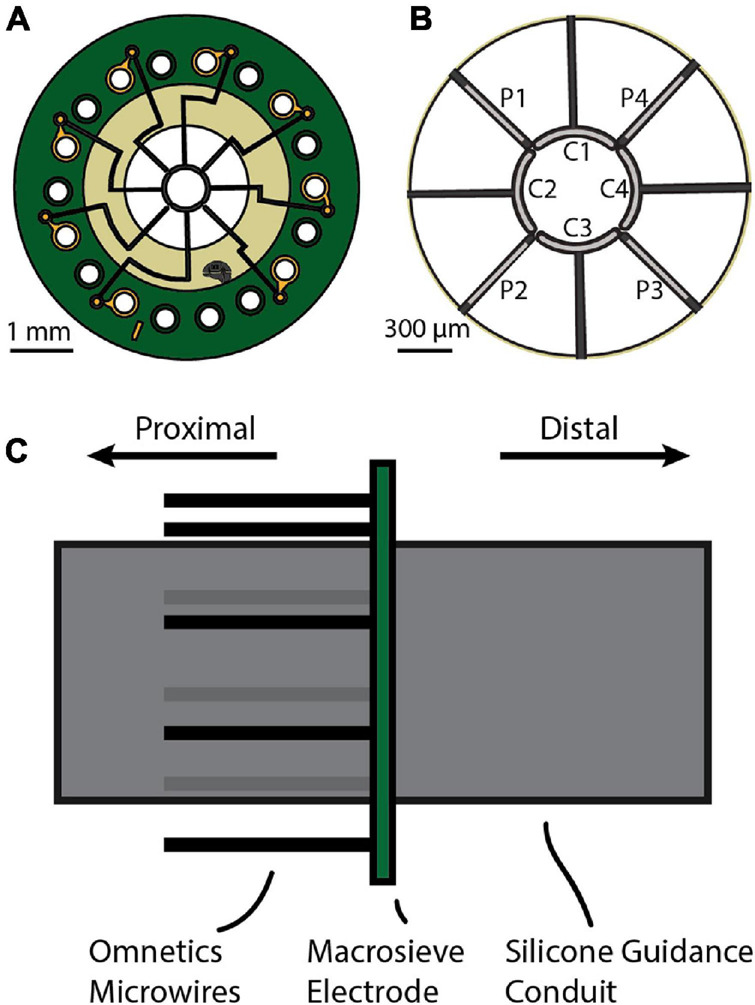
**(A)** The macro-sieve electrode (MSE) is a circular disc with three concentric rings: a central active region, a middle polyimide ring, and an outer PCB board. The active region has nine transit zones through which axons regenerate. It also has a circular hub and radiating spokes that together hold eight platinum-iridium leads that inject current into adjacent nerve tissue. The middle polyimide ring houses embedded traces that relay current to the leads from solderable through-holes located on the outer PCB ring. **(B)** Enlarged view of the active region. The circular hub and radiating spokes together define the boundaries of the nine transit zones. The circular hub houses four core leads (C1, C2, C3, and C4). Alternating radial spokes house four peripheral leads (P1, P2, P3, and P4). **(C)** Side view of the MSE assembly prior to implantation. Silicone guidance conduits affixed to either side of the polyimide ring will guide regenerating axons through the transit zones. Eight microwires from the attached Omnetics connector are soldered to the PCB’s contact pads to interface the leads with an external stimulator.

The macro-sieve electrode (MSE) maximizes functional recovery by eschewing a dense grid of small transit zones in favor of nine large transit zones with a combined area in excess of 2 mm^2^. The transit zones are bound by a circular hub and eight radiating spokes that together house eight platinum-iridium leads (Figure 1). Four “core” leads with a curved geometry lie on the circular hub (labeled C1, C2, C3, and C4). The remaining four “peripheral” leads lie on alternating radial spokes (labeled P1, P2, P3, and P4). With just eight leads, the MSE is capable of selective recruitment of axon clusters throughout the nerve’s cross-section by the coordinated application of cathodic and anodic currents (i.e., current steering). This sets it apart from other interfaces that seek greater selectivity by increasing the number of channels (e.g., Tan et al., 2014; Davis et al., 2016). The robust stability, close intimacy with target axons, and selective control afforded by the MSE’s regenerative design make it a promising aspirant for a sensory feedback interface. However, the transected and subsequently regenerated axons with which it interfaces differ markedly from undisrupted axons in both morphology and caliber distribution. Regenerated axons have thinner myelin sheaths, tend toward smaller calibers, and have shorter internodal separation than their undisrupted counterparts (Beuche and Friede, 1985; Friede and Beuche, 1985; Negredo et al., 2004; Castro et al., 2008), and so can be expected to differ in their electrophysiological response to stimulation by an implanted electrode interface (McNeal, 1976). Prior simulation work by our group (Zellmer et al., 2018) suggests that recruitment thresholds for regenerated axons are not inherently higher or lower than for undisrupted axons, but depend on their distance from the stimulating lead and the manner of stimulation. Accordingly, Zellmer et al. (2018) predict that regenerated axons located near the stimulating lead should have lower thresholds than undisrupted axons and that those further away should have higher thresholds. MacEwan et al. (2016) demonstrated selective recruitment of distal musculature using single-channel stimuli from an MSE implanted in the rat sciatic nerve, indicating its potential use as a chronic implant for the restoration of motor control to a paralyzed limb. *In vivo* measurement of detection thresholds is vital to the establishment of the MSE’s candidacy as an interface for delivering sensory feedback.

Testing of sensory feedback interfaces has traditionally relied on implantation of devices in a limited number of human subjects and surveying reported percepts arising from different stimulus conditions. However, absence of clinical approval hampers the gathering of essential data that can inform early stage development; animal models can provide a much-needed bridge during this phase. Rodent behavioral models have long been employed in the study of various sensory modalities (e.g., Laing et al., 1974; Kelly and Masterton, 1977; Uchida and Mainen, 2003; Gaese et al., 2006; Stuttgen et al., 2006; Butovas and Schwarz, 2007; Huber et al., 2008; Adibi and Arabzadeh, 2011; Mayrhofer et al., 2013). The present study developed a combined rat sciatic nerve and behavioral model to characterize the MSE’s performance as a sensory feedback interface. It implemented a go/no-go detection task to determine minimum current levels required to elicit a sensory response with 50% probability (i.e., the detection threshold) for various stimulus configurations across multiple timepoints.

## Materials and Methods

### Experimental Design

**FIGURE 2:**
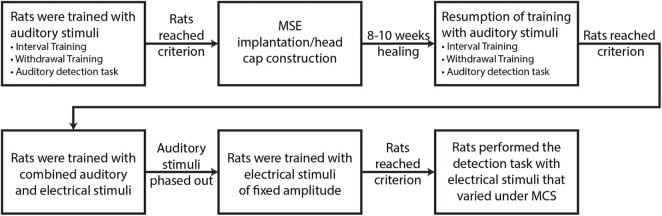
Main stages of experimental protocol.

Four food-restricted, male Lewis rats (Rats A, B, C, D; Charles River Laboratories, Wilmington, MA, United States) were studied on a behavioral task to measure current intensity detection thresholds for various MSE sector-activations of the sciatic nerve. The rats were trained with auditory stimuli prior to MSE implantation. They next proceeded to surgical implantation of an MSE in the right sciatic nerve and construction of a head cap with an embedded connector (Omnetics Connector Corporation, Minneapolis, MN, United States) for external interfacing. After 8–10 weeks of healing, the rats resumed training and transitioned to electrical stimuli under the method of constant stimuli (MCS), which applied stimulus intensities in random order from a pre-defined list without replacement. MCS was applied for nine different monopolar stimulus configurations in which the MSE stimulated the nerve and titanium screws embedded in the skull provided a return path for current stimuli. The first configuration passed equal currents through all eight channels simultaneously (i.e., multi-channel experiment), whereas the remaining eight configurations passed current through each channel individually (i.e., single-channel experiments). A flowchart representing the described protocol is presented in Figure 2. The data generated by this protocol enabled the derivation of psychometric curves depicting the probability of correct stimulus detections as a function of current intensity, and subsequent derivation of detection thresholds. All experimental procedures were conducted in accordance with regulations specified by the Institutional Animal Care and Use Committee at Washington University School of Medicine in St. Louis.

### Experimental Apparatus

**FIGURE 3:**
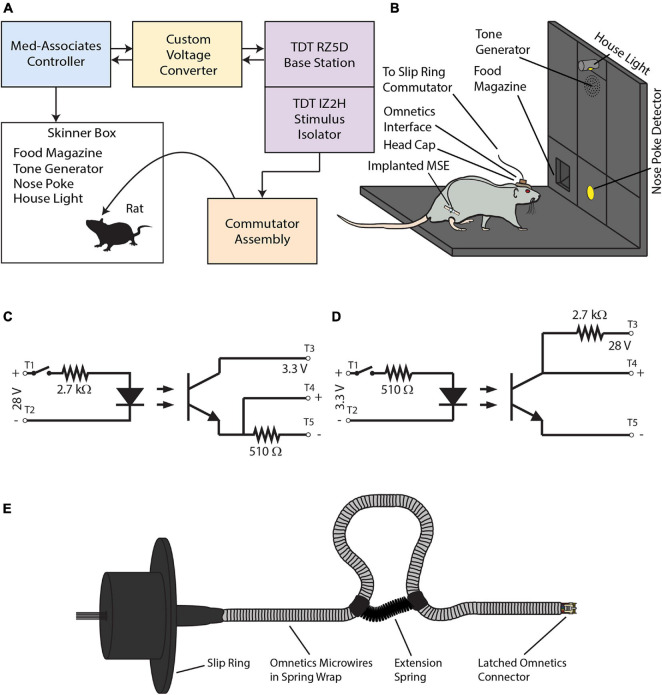
**(A)** The experimental apparatus consists of a behavioral module (blue panel) and an electrophysiological module (violet panels). A voltage converter (yellow panel) mediates TTL communications between the two modules. A slip-ring commutator assembly (orange panel) transmits electrical stimuli to the MSE implanted in the rat’s sciatic nerve via the skull-mounted Omnetics interface. **(B)** Diagram of the Skinner box. **(C)** Optical relay circuit used to step voltages down from 28 to 3.3 V. **(D)** Optical relay circuit used to step voltages up from 3.3 to 28 V. **(E)** The slip-ring commutator.

The experimental apparatus had four parts–a behavioral module, an electrophysiological module, a custom-built voltage converter for intermodular communications, and a commutator assembly (Figure 3A).

#### Behavioral Module

Rats performed the go/no-go task within the behavioral module (Med Associates, Inc., St. Albans, VT, United States). This consisted of a modular Skinner box (height 12″, depth 10″, width 12″) enclosed within a sound- and light-attenuating chamber. The panel on the Skinner box’s right side had three vertical bays (each of width 3″). The central bay housed (from top to bottom) a house light, a 2,900 Hz tone generator, and a nose-poke detector (diameter 1″; centered 2.5″ above floor) that detected snout insertions by means of an infrared beam detector. The left bay housed a rectangular food magazine (width 2¼″, height 1¾″; bottom edge was flush with floor) that was connected to a food pellet dispenser for 20-mg food pellets (BioServ, Prospect, CT, United States; #F0163). The right bay housed no instrumentation. An external, hand-held push button enabled manual interventions such as switching between or combining auditory and electrical stimuli, controlling the house light, or dispensing food pellets. Holes drilled in the Skinner box’s ceiling accommodated a webcam for remote monitoring of rat behavior and the commutator assembly. Figure 3B provides a detailed view of the Skinner box interior.

The behavioral module operated on a hardware-specific, state-based programming language called Med-State Notation (Med Associates, Inc., St. Albans, VT, United States). Programs written in this language executed available command sequences during “interrupts” that occurred every 5 ms. The nose-poke detector registered an ON signal for each interrupt in which the rat’s snout disrupted the detector’s infrared beam. For prolonged insertions, minor movements of the head, snout, or whiskers during insertion would sometimes disengage and reengage the infrared beam rapidly across successive interrupts, resulting in erroneous withdrawal registrations. To mitigate this effect, withdrawal registration was delayed by 30 interrupts (i.e., 150 ms) after the rat disengaged the infrared beam. Calculation of the true moment of withdrawal accounted for this delay.

#### Electrophysiological Module

The electrophysiological module (Tucker-Davis Technologies, Alachua, FL, United States) consisted of an RZ5D BioAmp Processor that interfaced with an IZ2H stimulus isolator powered by an LZ48-500M battery pack. The IZ2H transmitted electrical stimuli to the rat’s skull-mounted male connector and onward to the implanted MSE via the commutator assembly. The IZ2H interfaced with the commutator assembly via a DB26-to-DB25 DBF MiniDBM adapter (Tucker-Davis Technologies, Alachua, FL, United States).

The electrophysiological module operated on programs written in the OpenEx Software Suite (Tucker-Davis Technologies, Alachua, FL, United States) in conjunction with MATLAB (The Mathworks, Inc., Natick, MA, United States). An OpenEx program monitored the RZ5D’s DB25 channel bank for signals sent by the behavioral module via the voltage converter. These signals included a command to trigger the electrical stimulus to be delivered to the sciatic nerve, notifications of withdrawal, and notifications that the experimental session had started or ended. Each signal updated a corresponding index with the current system time. A parallel MATLAB script implementing MCS monitored these indices once every 10 ms, executing appropriate commands with each index change.

To sustain responding in the face of repeated presentations over successive trials of sub-threshold stimuli, the MATLAB script defined two modes of stimulus intensity selection. In “normal” mode, the script pulled intensities from the pre-defined MCS list. Failure to detect the stimulus over 1–3 consecutive trials caused the script to switch to “maintenance” mode, wherein the stimulus intensity was fixed at the lowest value known to elicit a visible muscle twitch in the right hind leg. The rat’s detection rate (DR) for such maintenance trials was at or near 100%. The script returned to normal mode when the rat successfully detected this supra-threshold stimulus over 1–2 consecutive trials.

#### Voltage Converter

The voltage converter enabled two-way communications between the behavioral and electrophysiological modules. It used optical relay circuits to step down 28-V signals outputted by the behavioral module to 3.3 V for input into the RZ5D’s digital I/O port. In the reverse direction, it stepped up 3.3-V signals outputted by the RZ5D to 28 V for input into the behavioral module. Figures 3C,D show the circuit schematics for stepping voltages from 28 to 3.3 V, and vice versa.

#### Commutator Assembly

The commutator assembly (Figure 3E) allowed the rat to move about the Skinner box without torsioning the wires. The commutator had a 12-channel slip ring (AdaFruit Industries, New York, NY, United States; #1196) soldered to a latched, female connector (Omnetics Connector Corporation, Minneapolis, MN, United States; #A76855-001). A flexible sheath of stainless steel (Tollman Spring Company, Bristol, CT, United States) enclosed the connector’s microwires and protected them from gnawing. A low-force extension spring (McMaster-Carr, Elmhurst, IL, United States; #9654K513), attached to the sheath with heat shrink tubing, formed a loop that absorbed excess slack and prevented entanglement as the rat moved about the Skinner box. The completed assembly was affixed such that the wires passed through a hole drilled through the Skinner box’s ceiling. During experiments, the suspended female connector connected with its male counterpart atop the rat’s skull, providing a means to send electrical signals from the IZ2H stimulus isolator to the sciatic nerve via the implanted MSE.

### Auditory Training and Behavioral Task Overview

#### Acclimation and Food Restriction

Rats were acclimated to the rodent housing facility, human handling, and finally the Skinner box. Acclimated rats received a pre-measured quantity of food each day, corresponding to how much they would typically eat in 1 h. Weights were monitored each weekday. Rats that dropped below 80% pre-restriction weight were returned to unlimited food access.

#### Training on Auditory Stimuli

Rats were trained with auditory stimuli prior to MSE implantation. Initially, their approach to the food magazine and nose-poke detector was shaped manually by monitoring behavior via a webcam and using the hand-held push button to deliver food pellets. An insertion into the nose-poke detector lasting a few milliseconds was sufficient to trigger the release of a food pellet. Next, the rats were shaped to insert their snouts into the detector for progressively longer intervals. Maintaining uninterrupted insertion for the prescribed interval counted as a successful response and triggered reinforcement accompanied by an auditory tone lasting 500 ms. Failure to maintain insertion resulted in a 7-s timeout with the house light switched off. The initial insertion interval was 0.5 s. The interval was incremented by 0.5 s after four consecutive successes to a maximum of 6 s. It was decremented by 0.5 s after three consecutive failures to a minimum of 0.5 s. This “interval training” stage lasted for up to six daily sessions of 1–2 h each. After learning to quickly reach and maintain the maximum insertion interval of 6 s, the rats progressed to the “withdrawal training” stage. Here, food-pellet release was no longer contingent on completing the required insertion, but rather on the time to withdraw following stimulus onset. The rats had 500 ms to withdraw following onset of the auditory stimulus triggered by successfully maintaining insertion over a fixed 3-s interval. Correct withdrawals (CWs), corresponding to successful detections, occurred within 500 ms of stimulus onset and resulted in reinforcement. Late withdrawals (LWs) more than 500 ms after stimulus onset represented failed detections and went unreinforced. Early withdrawals (EWs) before stimulus onset resulted in a 7-s timeout with the house light switched off and did not contribute to detection statistics. Two metrics to measure the rat’s overall performance were defined. The stimulation rate (SR) was the proportion of trials out of the total in which the rat was stimulated. The DR was the proportion of stimulated trials in which the rat correctly detected the stimulus. Mathematically,


(1) $$\mathrm{Stimulation}\mathrm{Rate}=\frac{\text{CW}+\text{LW}}{\text{EW}+\text{CW}+\text{LW}},$$

(2) $$\mathrm{Detection}\mathrm{Rate}=\frac{\text{CW}}{\text{CW}+\text{LW}}.$$

**FIGURE 4:**
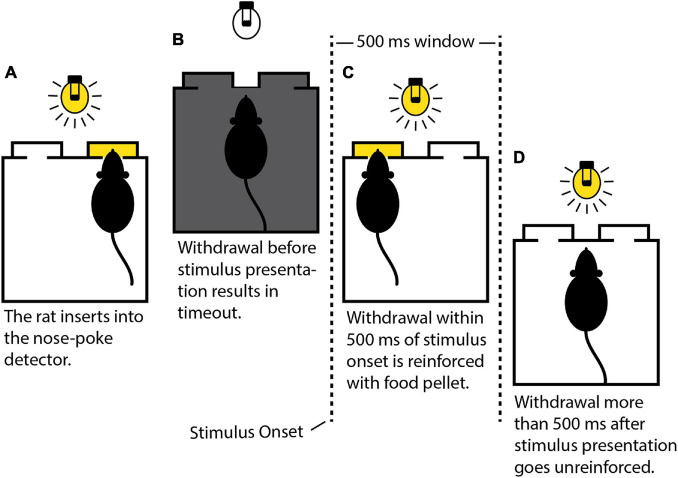
Schematic depiction of the behavioral task. **(A)** The rat must insert its snout into the nose-poke detector (right side) and maintain insertion uninterrupted until stimulus presentation. **(B)** Premature withdrawal results in a 7-s timeout during which the house light is extinguished. **(C)** Withdrawal within 500 ms of stimulus onset triggers the release of a food pellet (left side). **(D)** Withdrawal outside the 500-ms window results in a 3-s timeout without reinforcement.

Rats progressed to the main auditory detection task after achieving criterion on the withdrawal training task, i.e., DR ≥ 90% on two consecutive training days. The rats required up to five daily sessions of withdrawal training, lasting 1–2 h, before reaching criterion. The main task was identical to withdrawal training in all but one respect–instead of a fixed insertion interval across trials, insertion intervals were now randomized (3 ± 1.5 s, distributed uniformly). After the rats achieved criterion on the main task with a sufficiently high SR (SR ≥ 70%; 3–4 daily sessions, 1–2 h each), they proceeded to surgery after suspending food restriction and growing to 300+ g. Figure 4 summarizes the main behavioral task.

### Preparation of Macro-Sieve Assembly

A latched male connector (Omnetics Connector Corporation, Minneapolis, MN, United States; #A76854-001) was modified as follows. Wires for channels 1–6 were removed. Wires for channels 7–10 were cut to 2.5″ and stripped of all insulation. Wires for channels 11–18 were cut to 8.75″ and soldered at 290°C to the MSE’s eight through-holes using a lead-free alloy (96.5% Sn, 3% Ag, 0.5% Cu) and a no-clean flux (Chip Quick, Inc., Ancaster, ON, Canada; #SMD291). A pair of 4-mm silicone guidance conduits with inner diameter 2 mm (A-M Systems, Sequim, WA, United States; #808500) were affixed on either side of the MSE’s middle polyimide ring using a small quantity of medical grade silicone adhesive (Factor II, Lakeside, AZ, United States; #A-564). After curing for 24 h, additional Factor II adhesive was used to insulate the solder joints and impart mechanical support to the conduit/polyimide interface. After curing, this last step was repeated as needed until complete insulation was achieved. Finally, channel impedances were measured *in vitro* at 1 and 5 kHz using an Autolab PGSTAT128N potentiostat (Metrohm Autolab, Utrecht, Netherlands).

### Surgical Implantation of Macro-Sieve Electrode

Rats underwent surgery to implant the MSE in the right sciatic nerve and mount the attached connector atop the skull in a dental acrylic head cap. All surgical instruments and implants were sterilized by ethylene oxide or autoclave prior to surgery. Rats were anesthetized with isoflurane (administered IH, 4% induction, 2% maintenance) and injected with an analgesic for post-operative pain mediation (buprenorphine SR, administered SC, 1.2 mg/kg). The right hind leg, back, and scalp were shaved and sterilized using isopropyl alcohol and Betadine solution. Artificial tears applied with a cotton-tipped swab protected the eyes during surgery. The rats were head-fixed in a stereotaxic frame with a fitted nose cone to maintain anesthesia. The scalp was incised sagittally along the midline and cleaned of blood and soft tissue using a cotton-tipped swab dipped in hydrogen peroxide. Persistent bleeds were cauterized. Four to six holes were drilled into the skull using a #56-microdrill set in a hand-driven pin vise. Drilling stopped upon encountering resistance from the bone’s underlying cancellous layer. A 0–80 titanium hex screw was inserted into each hole to ensure that the head cap would remain firmly anchored to the skull.

**FIGURE 5:**
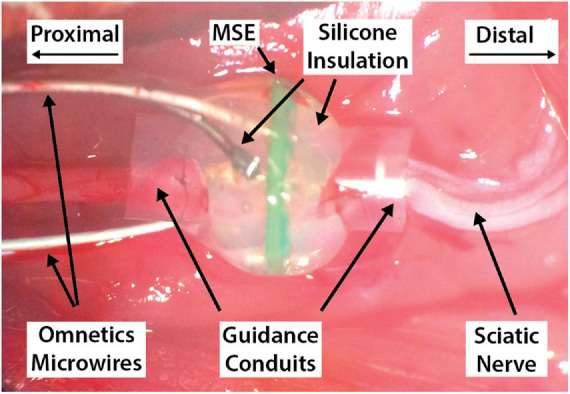
An implanted macro-sieve electrode, with proximal and distal nerve stumps sutured into their respective guidance conduits. The Omnetics connector and its trailing microwires have been routed dorsally through a subcutaneous tunnel and out through a sagittal scalp incision exposing the skull.

Next, the sciatic nerve was exposed via a dorsolateral gluteal muscle-splitting incision, and blunt dissection. Nerve transection 5 mm proximal to the sciatic trifurcation preceded suturing of nerve stumps into the MSE assembly’s silicone guidance conduits using #8 microsuture (Figure 5). Separation of skin from fascia with a pair of blunt-tipped forceps created a subcutaneous dorsal tunnel between the leg and scalp incisions, through which the connector (with trailing microwires) was passed up to the skull. The connector was wrapped in a protective layer of Parafilm (Bemis Company, Inc., Neenah, WI, United States) prior to sterilization to prevent contamination of its electrical contacts during this step. This was removed once the connector was positioned over the skull. Exposed wires for channels 7–10 were wrapped around nearby skull screws; these would provide a return path for applied current stimuli. The connector was placed within a custom cylindrical titanium chamber whose threaded outer walls allowed the placement of a protective Delrin plastic (DuPont, Wilmington, DE, United States) screw cap when the connector was not in use. Care was taken to ensure that the connector’s latching mechanism rested well above the chamber’s rim. The connector, titanium chamber, and wrapped wires were fixed in place by building a head cap with two UV-cured dental acrylics (this technique was based on Park et al., 2016). A base layer of self-bonding acrylic (Fusio; Henry Schein Inc., Melville, NY, United States) was first applied to all areas of exposed skull. After setting for 30 s, this was cured with a dental UV lamp for a further 30 s. The body of the head cap was then built up little by little using a second UV-cured dental acrylic that was not self-adhering and therefore less expensive (Flow-it; Henry Schein Inc., Melville, NY, United States). Each small application of Flow-it was cured separately for 30 s using the dental UV lamp.

With MSE implantation and head cap construction completed, all muscle and skin incisions were closed with 5-0 vicryl and 4-0 nylon suture, respectively. Rats were released from the stereotaxic frame and returned to their cages for post-operative monitoring. Cages were placed partially on top of a heating pad to create a temperature gradient for the rats’ comfort. Post-operative monitoring continued until suture removal at 7–10 days post-surgery.

### Resumption of Training, Data Collection

Rats resumed training with auditory stimuli 8–10 weeks post-surgery, progressing from interval training (2–5 days, 1–2 h each day) to withdrawal training (1–2 days, 1–2 h each day) and then reestablishment of criterion performance on the main auditory detection task (3–5 days, 1–2 h each day). They were next trained with combined auditory and electrical stimuli presented synchronously. Commencement of each experimental day with electrical stimulation was preceded by *in vivo* impedance measurements using the Synapse software suite (Tucker-Davis Technologies, Alachua, FL, United States). The electrical stimuli comprised equal currents passed through all eight MSE channels (i.e., multi-channel configuration). The IZ2H stimulus isolator was programmed to deliver 500 ms, 50 Hz biphasic pulse trains in which the leading and lagging phases each lasted 200 μs with no interphase gap. The stimulus intensity was of a fixed amplitude, chosen to elicit a visible twitch response in the leg and foot without any evident distress. The auditory stimuli were terminated mid-session to train responding to electrical stimuli alone. Following 2–4 days of these combined stimulus sessions in which the auditory stimuli were terminated mid-session and only electrical stimuli were presented in the latter part of the session, the rats progressed to sessions in which they were trained solely with fixed-intensity current stimuli, without any auditory stimuli, until they achieved criterion performance. Finally, the rats were trained with electrical stimuli whose amplitudes varied under MCS. Rats A, B, C, and D all performed MCS with the multi-channel stimulus configuration for up to 9 days of data collection, lasting up to 3 h per day. Only Rats C and D performed MCS with the single-channel stimulus configurations. For Rat C, data collection for each channel lasted 1–2 days with an average daily duration of 1¾ h (maximum duration: 3 h); one round of data collection was performed. For Rat D, data collection for each channel lasted 1–4 days. The average duration of data collection per day was roughly 3½ h (maximum duration: 6½ h). Two rounds of data collection were performed.

### Data Analysis

#### Processing the Data

Time-stamped event logs were used to populate a PostgreSQL database from which trial-level information for each dataset was extracted for analysis in MATLAB. Each trial had an associated stimulus amplitude, a withdrawal time following stimulus onset, an outcome (EW, CW, or LW), and a mode (normal or maintenance). EWs were discarded at the outset. Trials in which the rat withdrew less than 100 ms after stimulus onset were also excluded from analysis; CWs that occurred faster than this minimum reaction time were deemed to have been coincidental, and not to have been in response to the actual stimulus. Finally, maintenance trials were excluded unless noted otherwise.

#### Psychometric Model

The subsequent analysis and notation closely follows that of Wichmann and Hill (2001a, b). For MCS, each dataset had *K* stimulus amplitudes (or blocks) denoted by the vector *x*. The number of trials associated with each amplitude was denoted by the vector *n*, so that the total number of trials was $N=\sum_{i=1}^{K}n_{i}$. The proportion of correct detections for each stimulus amplitude was denoted by the vector *y*. A psychometric model was fitted to this empirical distribution of probabilities. The generalized psychometric model may be written as


(3) ψ(x;θ)=γ+(1-γ-λ)F(α,β).

Here, ψ is the probability of correct detection expressed as a function of stimulus intensity *x* and the parameter vector θ = {α,β,γ,λ}. The parameter α denotes the mean of the sigmoidal driving function *F*(*x*;α,β); the parameter β is the slope parameter (not to be confused with the driving function’s actual slope). The parameters γ and λ denote the guess rate and lapse rate, respectively. These latter two parameters are of secondary scientific interest as they emerge from the stimulus-independent mechanisms underlying guessing and lapsing (Wichmann and Hill, 2001a). The choice of driving function depends on the task design and is presumed to reflect the underlying detection mechanism (Wichmann and Hill, 2001a). This study used the Quick function


(4) $$F\left(x;\alpha ,\beta \right)=1-2^{\left(-{\left(\frac{x}{\alpha }\right)}^{\beta }\right)},$$

for which *F*(*x* = α) = 0.5 and *F*′(*x* = α) = βln(2)/2α. Note that the psychometric mean α corresponds to the 50% detection threshold *x_T* only when γ = λ = 0. Otherwise, *x_T* may be calculated by taking the inversion of Eq. 3. Likewise, the psychometric slope ψ′ (*x*_*T*_;θ) equals the slope of the driving function *F*′(*x* = α) only when γ = λ = 0. Otherwise, it may be calculated by taking the derivative of Eq. 3.

The psychometric model described by Eqs 4 and 5 was fitted to the empirical probability distribution *y* by using a bounded version of the Nelder-Mead simplex algorithm (D’Errico, 2021) to minimize the negative log-likelihood function (Wichmann and Hill, 2001a).


(5) l(θ;y)=-∑i=1K(log(niniyi)+yinilog⁢ψ(xi;θ)+(1-yi)nilog⁢[1-ψ(xi;θ)]).

Although γ was pegged to the rat’s detection performance level at *x* = 0A, λ was allowed to vary between 0 and 0.05.

#### Goodness of Fit

Goodness of fit was assessed using the Monte Carlo bootstrap (Wichmann and Hill, 2001a). The empirically derived psychometric model $\psi \left(x;\hat{\theta }\right)$ served as a generating function for 10,000 simulated datasets with the same stimulus amplitude distribution. The deviance metric *D* quantified the empirical probability distribution’s closeness to the fitted model. Deviance was calculated as


(6) $$D=2\sum_{i=1}^{K}\left\{n_{i}y_{i}\log\left(\frac{y_{i}}{\psi \left(x_{i};\theta \right)}\right)+n_{i}\left(1-y_{i}\right)\log\left(\frac{1-y_{i}}{1-\psi \left(x_{i};\theta \right)}\right)\right\}.$$

$\psi \left(x;\hat{\theta }\right)$ was considered a poor fit if $\hat{\text{D}}$ exceeded the 97.5th percentile of the simulated deviance distribution *D*^∗^, i.e., $\hat{\text{D}}>D^{*\left(97.5\right)}$. The fit was improved by identifying and removing outliers and then recalculating $\hat{\theta }$. Outliers were identified using the jackknife resampling technique (Wichmann and Hill, 2001a). For a dataset with *K* amplitudes *x*, the *i*th jackknife dataset *x*_(–*i*)_ was derived by removing the *i*th amplitude *x_i*. Parameter vectors ${\hat{\theta }}_{\left(-1\right)},{\hat{\theta }}_{\left(-2\right)},\ldots {\hat{\theta }}_{\left(-K\right)}$ corresponding to each jackknife were calculated, along with deviances *D*_(−1)_,*D*_(−2)_,…*D*_(−*K*)_. The *i*th amplitude *x_i* was considered an outlier if the corresponding reduction in deviance satisfied *D*−*D*_(−*i*)_ > 6.63.

#### Confidence Intervals

The 10,000 bootstrapped parameter fits ${\hat{\theta }}^{*}$ were also used to calculate bias-corrected and accelerated (BC_a_) 95% confidence intervals (CI_95_) for estimated threshold and slope parameters $\hat{\alpha }$ and $\hat{\beta }$ (Wichmann and Hill, 2001b). The ϵ-level confidence interval endpoint for some parameter $\hat{\rho }$ is calculated as


(7) $${\hat{\rho }}_{{\text{BC}}_{\text{a}}}\left(\epsilon \right)={\hat{G}}^{-1}\left(\text{CG}\left({\hat{z}}_{0}+\frac{{\hat{z}}_{0}+z^{\left(\epsilon \right)}}{1-\hat{a}\left({\hat{z}}_{0}+z^{\left(\epsilon \right)}\right)}\right)\right).$$

Here, ${\hat{\text{G}}}^{-1}$ is the inverse cumulative distribution function of the bootstrap simulations ${\hat{\rho }}^{*}$. CG is the cumulative Gaussian distribution function. ${\hat{z}}_{0}$ and $\hat{a}$ are the bias-correction and acceleration terms, respectively (details of this calculation may be found in Efron and Tibshirani’s (1993) *Introduction to the Bootstrap*).

#### Comparison of Thresholds and Slopes for Core and Peripheral Channels

Due to the limited data available for Rat C and Rat D, all available single-channel detection thresholds (both rats, all rounds of data collection) were pooled into two groups–core and peripheral. Comparison of detection thresholds between these groups proceeded on the basis of the Mann–Whitney *U* test, with *p* < 0.05 accepted as a significant difference. An equivalent analysis for slopes was also performed.

## Results

MCS was used to generate psychometric curves for multi-channel and single-channel stimulus configurations. Fitted psychometric parameters θ = {α,β,γ,λ} were used to calculate detection thresholds *x_T* and psychometric slopes ψ′ (*x*_*T*_,θ) using the inversion and derivative of Eq. 3, respectively. Table 1 summarizes the data collected.

**TABLE 1:** Summary of data collected.

	Multi-channel stimulation	Single-channel stimulation							
		C1	C2	C3	C4	P1	P2	P3	P4
Rat A	•								
Rat B	•								
Rat C	•	•	•		•	•	•	•	•
Rat D	•	••	••	••	••	••	•	•	

*Each dot represents one dataset.*

*Pairs of dots indicate data collected at two-timepoints (longitudinal data).*

*Single-channel stimulation by channel C3 in Rat C and channel P4 in Rat D produced no leg twitch or behavioral response up to 200 μA, precluding data collection and subsequent generation of psychometric curves for these channels.*

### Multi-Channel Stimulation

**FIGURE 6:**
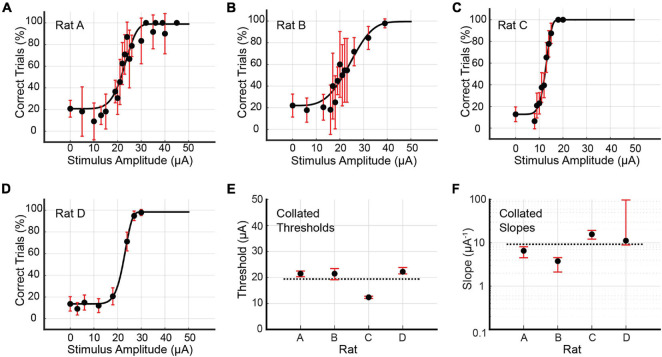
**(A–D)** Psychometric curves for Rats A, B, C, and D stimulated with the multi-channel configuration, in which equal currents passed through all eight channels simultaneously. Reported stimulus amplitudes correspond to currents passed through each individual channel, and not the total current. Error bars represent 95% confidence intervals for binomial distributions based on detection probabilities. See Table 2 for fitted parameters, 50% detection thresholds, slopes, and goodness of fit metrics. **(E,F)** Collated 50% detection thresholds (*x_T*) and slopes (ψ′ (*x*_*T*_)) extracted from the preceding four psychometric curves. Horizontal dotted lines represent the mean values across all rats. Error bars represent 95% BC_a_ confidence intervals.

Psychometric curves for the multi-channel stimulus configuration were generated for Rats A, B, C, and D (see Figure 6 and Table 2). Rat A was stimulated at 21 unique amplitudes over nine sessions spanning 25 days (114–138 days post-implantation). The number of trials per amplitude varied from 10 to 106 trials. For Rat B, 14 unique stimulus amplitudes were used (10–59 trials). Data was gathered in five sessions spread over 8 days (119–126 days post-implantation). Rat C’s data collection occurred over eight sessions spanning 21 days (82–102 days post-implantation). The rat was stimulated at 13 unique amplitudes (18–94 trials). Rat D was stimulated at just eight unique amplitudes, with little variation in the number of trials per amplitude (98–103 trials). Data collection for Rat D took place over six sessions spanning 5 days (76–80 days post-implantation). For this stimulus configuration, the average threshold current per channel necessary to elicit a behavioral response was 19.37 μA (3.87 nC), and the average slope was 9.30 μA^–1^ (46.50 nC^–1^). Bootstrapped estimates of the thresholds’ CI_95_ widths ranged from 0.87 to 4.34 μA (0.17–0.87 nC). For slopes, CI_95_ widths for Rats A, B, and C were 3.60, 2.43, and 7.16 μA^–1^ (18.03, 12.13, and 35.79 nC^–1^), respectively. Rat D’s CI_95_ for slope was much larger, spanning 88.57 μA^–1^ (442.84 nC^–1^).

**TABLE 2:** Fitted parameters α, β, γ and λ for the four psychometric curves generated by multi-channel stimulation of Rats A, B, C, and D (see Figure 6).

	Days	*N*	α(μA)	β	γ	λ	*x*_*T*_(μA) (nC)	ψ′(*x*_*T*_;θ) (μA^−1^) (nC^−1^)	pDev
Rat A	114–138	687	22.87	6.12	20.75%	1.08%	21.46 ± 1.02/1.15 (4.29 ± 0.20/0.23)	6.54 ± 1.59/2.01 (32.71 ± 7.96/10.07)	0.93
Rat B	119–126	421	24.19	3.64	22.03%	0.48%	21.45 ± 1.99/2.35 (4.29 ± 0.40/0.47)	3.76 ± 0.79/1.64 (18.82 ± 3.94/8.19)	0.11
Rat C	82–102	555	12.76	6.98	12.77%	0.00%	12.37 ± 0.45/0.42 (2.47 ± 0.09/0.08)	15.71 ± 3.55/3.61 (78.57 ± 17.76/18.03)	0.49
Rat D	76–80	805	22.73	9.14	13.59%	1.57%	22.21 ± 1.61/0.85 (4.44 ± 0.32/0.17)	11.18 ± 86.28/2.29 (55.88 ± 431.40/11.44)	0.17

*Also presented are the total number of trials *N* (excluding outliers), the 50% detection threshold *x_T* and slope ψ′ (*x*_*T*_;θ), their 95% confidence intervals (expressed as upper bound/lower bound), and the goodness of fit measure pDev.*

### Single-Channel Stimulation

**FIGURE 7:**
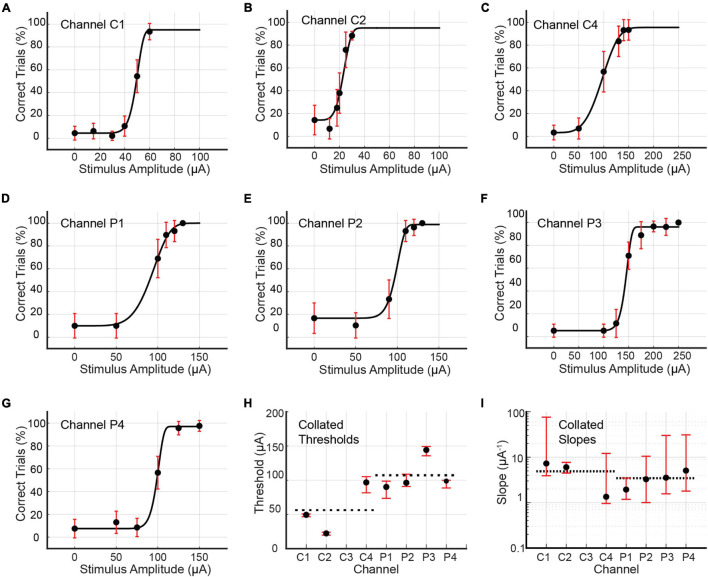
**(A–G)** Psychometric curves for Rat C generated by single-channel stimulation through individual channels C1, C2, C4, P1, P2, P3, and P4. Single-channel stimulation by channel C3 elicited no leg twitch or behavioral response up to 200 μA, precluding the generation of a psychometric curve for this channel. Error bars represent 95% confidence intervals for binomial distribution based on detection probability. See Table 3 for fitted parameters, 50% detection thresholds, slopes, and goodness of fit metrics. **(H,I)** Collated 50% detection thresholds (*x_T*) and slopes (ψ(xT′)) extracted from the preceding seven psychometric curves. Horizontal dotted lines depict mean values for core and peripheral channels. Error bars represent 95% BC_a_ confidence intervals.

Psychometric curves for single-channel stimulus configurations were generated for Rats C and D only. Single-channel stimulation of Rat C yielded seven psychometric curves, one each for channels C1, C2, C4, P1, P2, P3, and P4 (see Figure 7 and Table 3). Stimulation by C3 up to 200 μA produced no visible leg movement or behavioral response, precluding the generation of a psychometric curve for this channel. The remaining channels each stimulated the rat at six unique stimulus amplitudes, with the exception of P3, which used eight amplitudes. The distribution of trials among amplitudes was roughly uniform for each channel (approximately 30–60 trials per amplitude), although the dataset for C2 included 281 maintenance trials at 30 μA. Detection thresholds calculated for the three functioning core channels (C1, C2, and C4) ranged from 22.32 to 96.76 μA (4.64 to 19.35 nC; average value 56.17 μA or 11.21 nC), with CI_95_ widths between 3.95 and 23.53 μA (0.79 and 4.71 nC). Corresponding slopes ranged from 1.35 to 7.27 μA^–1^ (6.73–36.37 nC^–1^; average value 4.88 μA^–1^ or 24.39 nC^–1^), with CI_95_ widths from 3.26 to 72.17 μA^–1^ (16.28–431.40 nC^–1^). For the peripheral channels (P1, P2, P3, and P4), detection thresholds ranged from 90.18 to 144.20 μA (18.04–28.84 nC; average value 107.35 μA or 21.47 nC), with CI_95_ widths between 11.26 and 25.09 μA (2.25 and 5.02 nC). The corresponding slopes varied from 1.94 to 5.07 μA^–1^ (9.70–25.37 nC^–1^; average value 3.46 μA^–1^ or 17.29 nC^–1^), with CI_95_ widths between 2.31 and 29.15 μA^–1^ (11.56–145.75 nC^–1^).

**TABLE 3:** Fitted parameters α, β, γ, and λ for psychometric curves generated by single-channel stimulation of channels C1, C2, C4, P1, P2, P3, and P4 in Rat C (see Figure 7).

Channel	Days	*N*	α (μA)	β	γ	λ	*x*_*T*_(μA) (nC)	ψ′(*x*_*T*_;θ) (μA^−1^) (nC^−1^)	pDev
C1	149–151	280	49.38	11.42	4.44%	5.00%	49.42 ± 1.23/2.72 (9.88 ± 0.25/0.54)	7.27 ± 68.82/3.35 (36.37 ± 344.10/16.74)	0.23
C2	160	425 (180)	23.08	5.11	14.29%	5.00%	22.32 ± 1.80/2.53 (4.46 ± 0.36/0.51)	6.02 ± 1.71/1.55 (30.08 ± 8.55/7.73)	0.68
C4	161	178	96.32	4.05	3.33%	4.51%	96.76 ± 8.46/15.07 (19.35 ± 1.69/3.01)	1.35 ± 10.77/0.39 (6.73 ± 53.86/1.94)	0.09
P1	164	175	92.71	5.95	10.00%	0.00%	90.18 ± 8.41/16.68 (18.04 ± 1.68/3.34)	1.94 ± 1.55/0.76 (9.70 ± 7.75/3.80)	0.28
P2	165	175	98.51	12.41	16.67%	1.22%	96.26 ± 12.59/5.46 (19.25 ± 2.52/1.09)	3.27 ± 7.22/2.27 (16.37 ± 36.11/11.34)	0.56
P3	167–168	335	144.38	16.30	5.17%	3.89%	144.20 ± 5.18/8.49 (28.84 ± 1.04/1.70)	3.54 ± 26.70/1.98 (17.70 ± 133.51/9.90)	0.45
P4	169	266	99.18	16.54	7.50%	2.98%	98.74 ± 1.26/9.99 (19.75 ± 0.25/2.00)	5.07 ± 25.87/3.28 (25.37 ± 129.37/16.38)	0.33

*Also presented are the total number of trials *N* (excluding outliers), the 50% detection threshold *x_T* and slope ψ′ (*x*_*T*_;θ), their 95% confidence intervals (expressed as upper bound/lower bound), and the goodness of fit measure pDev. The data underlying channel C2’s psychometric curve included a relatively large number of maintenance trials. For this channel, the number of non-maintenance (i.e., normal) trials is reported in parentheses.*

**FIGURE 8:**
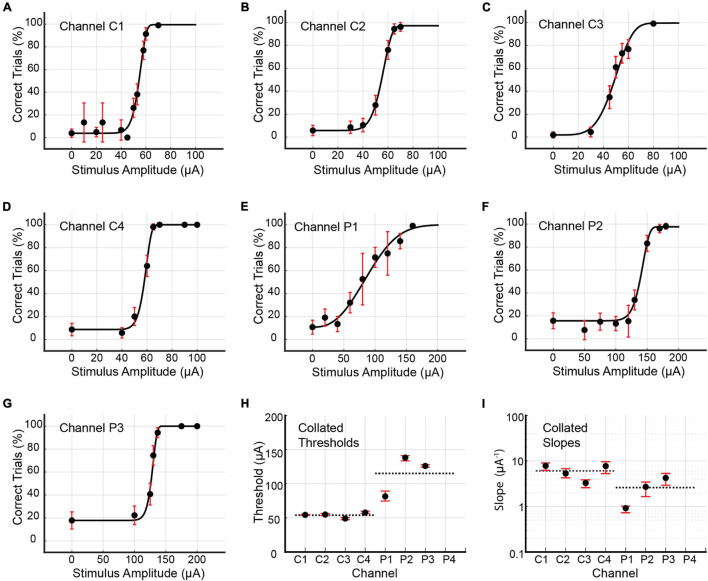
**(A–G)** Psychometric curves for Rat D generated by single-channel stimulation through individual channels C1, C2, C3, C4, P1, P2, and P3. Single-channel stimulation by channel P4 elicited no leg twitch or behavioral response up to 200 μA, precluding the generation of a psychometric curve for this channel. Error bars represent 95% confidence intervals for binomial distribution based on detection probability. See Table 4 for fitted parameters, 50% detection thresholds, slopes, and goodness of fit metrics. **(H,I)** Collated 50% detection thresholds (*x_T*) and slopes (ψ′ (*x*_*T*_)) extracted from the preceding seven psychometric curves. Horizontal dotted lines depict mean thresholds for core and peripheral channels. Error bars represent 95% BC_a_ confidence intervals.

Rat D underwent two rounds of data collection for single-channel stimuli. The first round resulted in the generation of seven psychometric curves for channels C1, C2, C3, C4, P1, P2, and P3 (see Figure 8 and Table 4). Stimulation by P4 produced no muscle twitch or behavioral response up to 200 μA, preventing psychometric curve generation for this channel. In the first round, the core channels (C1, C2, C3, and C4) had detection thresholds ranging from 49.05 to 57.75 μA (9.81–11.55 nC; average value 54.00 μA or 10.80 nC), with CI_95_ widths between 1.58 and 3.28 μA (0.32 and 0.66 nC). Corresponding slopes were between 3.29 and 7.82 μA^–1^ (16.44 and 39.10 nC^–1^; average value 6.04 μA^–1^ or 30.18 nC^–1^), with CI_95_ widths from 1.26 to 4.35 μA^–1^ (6.29–21.77 nC^–1^). The 3 functioning peripheral channels (P1, P2, and P3) had detection thresholds between 81.47 and 138.15 μA (16.29 and 27.63 nC; average value 115.20 μA or 23.04 nC), with CI_95_ widths from 3.72 to 14.53 μA (0.74–2.91 nC). The corresponding slopes ranged from 0.92 to 4.23 μA^–1^ (4.59–21.14 nC^–1^; average value 2.61 μA^–1^ or 13.07 nC^–1^), with CI_95_ widths between 0.29 and 2.40 μA^–1^ (1.43 and 11.99 nC^–1^).

**TABLE 4:** Fitted parameters α, β, γ, and λ for psychometric curves generated by single-channel stimulation of channels C1, C2, C3, C4, P1, P2, and P3 in Rat D.

Channel	Round	Days	*N*	α(μA)	β	γ	λ	*x*_*T*_(μA) (nC)	ψ′(*x*_*T*_;θ) (μA^−1^) (nC^−1^)	pDev
C1	R1	96–97	798	54.59	13.04	3.85%	0.45%	54.38 ± 0.68/0.90 (10.88 ± 0.14/0.18)	7.82 ± 1.22/1.60 (39.10 ± 6.08/7.98)	0.57
	R2	113–114	873	44.14	21.02	16.49%	1.58%	43.57 ± 0.66/0.59 (8.71 ± 0.13/0.12)	12.29 ± 3.70/2.52 (61.44 ± 18.48/12.59)	0.91
C2	R1	98	728	55.09	9.34	5.77%	2.92%	54.82 ± 1.53/1.52 (10.96 ± 0.31/0.30)	5.31 ± 1.45/1.06 (26.55 ± 7.26/5.31)	0.26
	R2	114–115	421	37.05	6.41	20.00%	4.90%	35.32 ± 1.84/2.81 (7.06 ± 0.37/0.56)	4.18 ± 1.80/1.25 (20.88 ± 8.99/6.25)	0.20
C3	R1	99–100	693	49.25	4.80	1.92%	0.59%	49.05 ± 1.44/1.84 (9.81 ± 0.29/0.37)	3.29 ± 0.57/0.69 (16.44 ± 2.84/3.46)	0.94
	R2	119–120	593	52.96	19.43	13.41%	3.70%	52.49 ± 1.06/1.16 (10.50 ± 0.21/0.23)	9.98 ± 9.11/2.98 (49.91 ± 45.53/14.88)	0.36
C4	R1	100–101	656	58.30	14.81	8.65%	0.00%	57.75 ± 2.02/1.17 (11.55 ± 0.40/0.23)	7.73 ± 1.90/2.46 (38.63 ± 9.49/12.28)	0.74
	R2	121	577	101.53	8.72	16.00%	0.71%	98.34 ± 2.57/3.05 (19.67 ± 0.51/0.61)	2.29 ± 0.45/0.67 (11.47 ± 2.26/3.36)	0.76
P1	R1	102–103	772	87.34	2.59	10.78%	0.00%	81.47 ± 7.77/6.76 (16.29 ± 1.55/1.35)	0.92 ± 0.10/0.19 (4.59 ± 0.49/0.94)	0.94
	R2	126–127	744	149.94	5.37	15.66%	0.58%	142.51 ± 5.32/5.97 (28.50 ± 1.06/1.19)	0.98 ± 0.15/0.20 (4.92 ± 0.75/0.98)	0.12
P2	R1	104–107	809	140.53	14.41	15.74%	2.34%	138.15 ± 3.06/4.71 (27.63 ± 0.61/0.94)	2.69 ± 0.75/1.04 (13.47 ± 3.73/5.19)	0.33
	R2		–	–	–	–	–	–	–	–
P3	R1	110–112	724	127.95	21.44	17.82%	0.00%	125.98 ± 1.94/1.78 (25.20 ± 0.39/0.36)	4.23 ± 1.10/1.30 (21.14 ± 5.49/6.50)	0.77
	R2		–	–	–	–	–	–	–	–

*For channels C1, C2, C3, C4, and P1, two sets of data are presented. These correspond to two rounds of data collection (R1 and R2) separated by 3 weeks. The seven psychometric curves of Figure 8 correspond to the first round of data collection (R1) for each channel. Also presented here are the total number of trials *N* (excluding outliers), the 50% detection threshold *x_T* and slope ψ(xT,θ)′, their 95% confidence intervals (expressed as upper bound/lower bound), and the goodness of fit measure pDev.*

**FIGURE 9:**
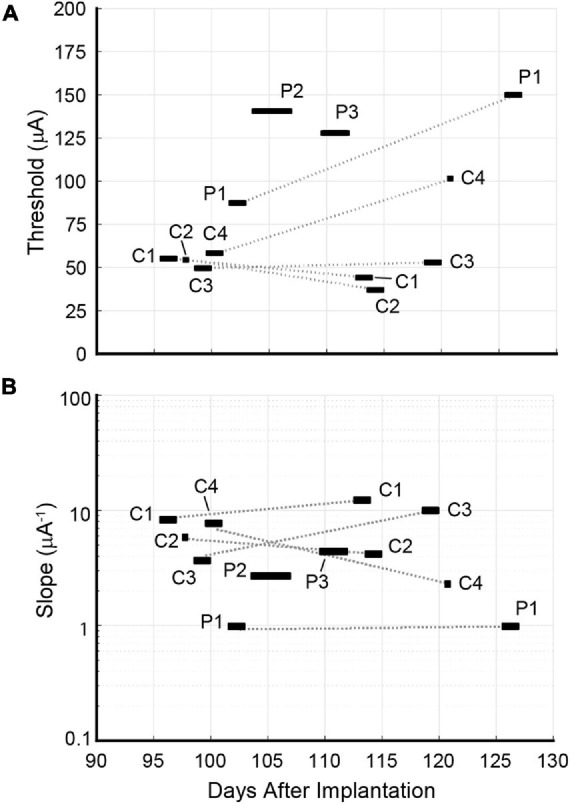
Longitudinal development of **(A)** detection thresholds x_T_ and **(B)** slopes ψ′(x_T_) for Rat D. Psychometric data for individual channels C1, C2, C3, C4, and P1 were calculated using behavioral data gathered at two timepoints spaced 3 weeks apart. The rat’s waning performance beyond 127 days post-implantation precluded a 2nd round of data collection for channels P2 and P3. Detection thresholds for channels C1 and C2 remained steady or decreased slightly across timepoints. C3’s detection threshold rose slightly. Detection thresholds for C4 and P1 increased dramatically. No pattern was discerned for the longitudinal development of slopes.

Data collection for the second round occurred approximately 3 weeks after the first and yielded a second set of psychometric curves for channels C1, C2, C3, C4, and P1. Channels P2 and P3 were excluded because the rat’s waning performance lowered the SR (see Eq. 1) and prevented the accumulation of sufficient trials for analysis. Detection thresholds for C1 and C2 decreased by 20 and 36%, respectively. While C3’s threshold increased marginally by 7%, thresholds for C4 and P1 increased markedly by 70 and 75%, respectively. The longitudinal development of thresholds and slopes is presented in Figure 9.

Mann–Whitney *U* tests comparing core and peripheral channels’ detection thresholds and slopes yielded *p*-values of 0.0008 and 0.02, respectively. This indicated that the two groups differed significantly on both parameters.

## Discussion

The complex and irreversible nature of MSE implantation has precluded testing in a human model during this early development phase. Nevertheless, there remains an urgent need for characterization of the MSE’s sensory performance in a preclinical setting to set the stage for human studies. Previous work by MacEwan et al. (2016) has shown that the rat sciatic nerve can successfully regenerate through the MSE’s nine transit zones. These factors together motivated the development of the combined rat sciatic nerve and behavioral model and its application to the measurement of MSE detection thresholds. Psychometric curves generated by MCS yielded a range of thresholds and slopes for multi-channel and single-channel stimulus configurations. Notably, per-channel current requirements for a given rat’s detection of multi-channel stimuli were lower than corresponding single-channel thresholds. Moreover, average current detection thresholds for the core channels were approximately half those of the peripheral channels (Mann–Whitney *U* test, *p* < 0.0008). The sections that follow discuss these findings in greater detail.

### Detection Thresholds for Multi-Channel Stimulus Configuration

Under multi-channel stimulation, Rats A, B, and D had similar detection thresholds of 21–22 μA per channel (corresponding to 4.2–4.4 nC of charge injection per channel, or a charge density of 157–163 nC/mm^2^). Rat C had a considerably lower threshold of 12 μA (2.4 nC, 91 nC/mm^2^). Notably, the threshold currents injected per channel for Rat C (12 μA) and Rat D (22 μA) fell well below their corresponding lowest single-channel thresholds of 22 μA (channel C2) and 49 μA (channel C3, first round), respectively. This can be explained by assuming that the axons activated by these single channels must have been the first to be activated under multi-channel stimulation. Since axon activation depends on local current density, the addition of equal currents from seven other channels likely lowered the per-channel current requirement to achieve the current density required for activation.

### Detection Thresholds for Single-Channel Stimulus Configurations

For single-channel stimulation, the average detection threshold for the core channels was approximately half that of the peripheral channels (Mann–Whitney *U* test, *p* < 0.0008); this relationship held true for both Rat C (56 μA vs. 107 μA, or 11.2 nC vs. 21.5 nC) and Rat D (56 μA vs. 115 μA, or 11.2 nC vs. 23.0 nC). In terms of current density, the discrepancy was even more pronounced: 351 nC/mm^2^ vs. 954 nC/mm^2^ for Rat C; 348 nC/mm^2^ vs. 1,085 nC/mm^2^ for Rat D. The divergence of core and peripheral channel thresholds may have had multiple causes.

Following transection, peripheral nerve regeneration commences with the formation of a bridge of dense extracellular matrix and inflammatory cells between the proximal and distal stumps. Vascularization of this intervening tissue creates a pathway for migrating Schwann cells to tow proximal axons toward distal targets (Cattin and Lloyd, 2016). Suzuki et al. (1998) has shown that regeneration of sensory axons precedes that of motor axons immediately after axotomy. Accordingly, there is an increased proliferation of sensory axons toward the nerve’s center and of motor axons toward the periphery (Negredo et al., 2004; Lago et al., 2005). This differential proliferation implies that the distance from a core channel to an average regenerated sensory axon should be less than from a peripheral channel. Previous simulations by our group (Zellmer et al., 2018) have predicted that regenerated axons’ thresholds for activation are not inherently higher or lower than those of undisrupted axons, but rather depend on proximity to the stimulating lead. Effectively, thresholds for nearby regenerated axons should be lower than for naïve axons of the same caliber, while thresholds for regenerated axons that are farther away should be higher than for their naïve counterparts. Thus, the higher density of regenerated axons at the nerve’s center, coupled with the pronounced dependence of regenerated axons’ recruitment on lead proximity, may have contributed to the observed discrepancy between core and peripheral channels’ detection thresholds.

Lead geometry may also have played a role. The core channels (area: 32,000 μm^2^ each) are curved, while the peripheral channels (area: 22,500 μm^2^ each) are straight. The core channels’ greater areas means that their current density for a given current level is lower than the corresponding current density for the peripheral channels. Generally, higher current density is associated with increased axon recruitment. Concurrently, the core channels’ curved geometry means that their “centers of mass” lie closer to centrally located axons than if their geometry was straight. Thus, the core channels may have had an outsized effect on central axons despite their larger areas, pushing their thresholds down. This effect would be magnified by the concentration of sensory axons toward the nerve’s center.

The peripheral channels’ radial placement is also of concern. These channels extend to a radius of 850 μm from the active region’s center, far exceeding the 1-mm diameter of regenerated rat sciatic nerve (MacEwan et al., 2016), and even the 1.4-mm diameter of undisrupted nerve (Tyler and Durand, 2003). This suggests that some fraction of the peripheral channels’ currents was injected into the extraneural space. However, the enclosure of the active region within a pair of 4-mm silicone guidance conduits would have ensured that any extraneural current must have flowed along the nerve’s periphery, contributing to axon recruitment. It remains unclear to what extent the peripheral channels extruded from the regenerated nerve, and how much their extrusion affected the detection threshold.

### Longitudinal Trends for Detection Thresholds

Longitudinal examination of Rat D’s detection thresholds using single-channel stimulation revealed disparate trends. Five channels (C1, C2, C3, C4, and P1) were measured at two timepoints 3 weeks apart. The decline in C1’s and C2’s thresholds may have been caused by the ongoing maturation of nearby regenerated axons (increased calibers, thicker myelin sheaths, and fewer unmyelinated axons), which is known to continue for up to 7 months post-implantation (Ceballos et al., 2002). Channel C3’s threshold was stable, showing a minor increase between the two rounds. The dramatic rise in C4 and P1’s thresholds coincided with increased channel impedances. For C4, the first round of data was gathered 100–101 days post-implantation. Corresponding impedances at 1 kHz ranged between 62.28 and 94.45 kΩ. From the 119th day onward, C4’s impedance became erratic. Measured values on this day ranged between 26.74 and 397.08 kΩ. The second round of data was gathered on the 121st day. Although the impedances measured on this day were lower than 2 days prior, on subsequent days (up to 127 days post-implantation) some measurements exceeded 1 MΩ. For P1, the first and second rounds of data were gathered 102–103 and 126–127 days post-implantation, respectively. The corresponding impedances for these two rounds ranged between 86.16 and 166.66 kΩ, and between 161.34 and 771.69 kΩ, respectively. Impedances for P2 and P3 also rose drastically after their respective first rounds of data collection, which may explain why the rat’s performance degraded during the attempted second round of data collection for these channels. Such sudden degradation of impedance suggests failure of the lead, the solder joint, the associated microwire, or damage to the skull-mounted connector. It remains unclear where the failure occurred.

### Psychometric Slopes

The psychometric slope ψ(xT;θ)′ signifies the rapidity with which the percentagewise probability of stimulus detection rises with stimulus intensity. Measured slopes for multi-channel stimulation ranged from 3.76 to 15.71 μA^–1^ per channel, and for single-channel stimulation from 0.92 to 12.29 μA^–1^. A steep slope implies that activation of nearby axons occurs with sufficient reliability that the transition from low to high detection probability occurs over a short span of increasing current; a shallow slope implies the opposite. For both Rats C and D, single-channel stimulation yielded steeper average slopes for the core channels than the peripheral channels (Mann–Whitney *U* test, *p* < 0.02). This may reflect the differential proliferation of sensory axons described by Suzuki et al. (1998). Since core channels reside in a region of high axon density, small current increments should significantly increase the number of axons recruited and hence the probability of a behavioral response. Conversely, the lower density of axons surrounding the peripheral channels means that small current increments should recruit fewer additional axons, producing little change in the behavioral response probability.

### Longitudinal Trends for Psychometric Slopes

No clear longitudinal trends for Rat D’s slope values were discerned. Five channels (C1, C2, C3, C4, and P1) were measured across two timepoints. Two channels’ slopes became markedly steeper (C1 and C3 by 57 and 203%, respectively). One channel’s slope increased slightly (P1 by 7%). The remaining two channels’ slopes became shallower (C2 and C4 by 21 and 70%, respectively). There was no consistent relationship between changes in threshold and changes in slope.

### Confidence Intervals

Wichmann and Hill (2001b) stress that bootstrapped confidence intervals do not measure a parameter’s underlying variability, but rather the variability inherent in the sampling scheme (i.e., the selection of current amplitudes), the number of trials for each amplitude, and interactions between the sampling scheme and parameter calculation. In the present study, the relation between choice of sampling scheme and CI_95_ widths was readily apparent, as datasets whose sampling schemes placed fewer amplitudes in the sloped domain of the psychometric curve showed greater variation in bootstrapped parameter values and hence wider confidence intervals. This effect was more pronounced for slopes than thresholds. Bootstrapped slope values for Rat D under multi-channel stimulation showed considerable variation due to the placement of only one amplitude (24 μA) in the psychometric curve’s sloped domain (see Figures 6D,F). Similarly, bootstrapped slopes for Rat C under single-channel stimulation also varied considerably with the exception of channel C2, for which multiple amplitudes lay in the sloped domain (see Figures 7B,I). Bootstrapped thresholds also showed a degree of variation for some channels. In contrast, Rat D’s threshold and slope confidence intervals for single-channel stimulation were more constrained (see Figure 8), reflecting the adoption of a pseudo-adaptive strategy that evaluated the psychometric curve at multiple mid-session timepoints to determine whether the sampling scheme’s placement of amplitudes located them optimally in the curve’s sloped domain, so that more amplitudes could be added as necessary.

### Future Directions

Future experiments will generate high-resolution maps of detection thresholds across the nerve using multipolar stimulus configurations, in which the coordinated application of cathodic and anodic currents across multiple MSE channels will restrict recruitment to spatially distinct axon clusters (i.e., current steering). Likewise, given the likely non-uniform distribution of regenerated sensory axons across the implant cross-section (i.e., higher density in the central region), the MSE’s transit zones could be redesigned to be smaller and more numerous in the central region while remaining larger toward the periphery. This might enable more even recruitment of sensory axons by matching recruitment slopes across the cross-section. Additional simulation work is also needed to understand the interplay of non-uniform axon distribution, lead geometry, and peripheral channel extrusion on axon recruitment.

The use of adaptive algorithms in place of MCS will reduce the number of trials required for reliable threshold estimation, enabling a more robust longitudinal analysis of threshold stability. Additionally, the present go/no-go detection task can be adapted to a 2-alternative forced-choice paradigm to assess whether such selective axon cluster recruitment elicits sensory percepts whose perceived locations in the phantom limb are discriminable.

The methods presented in this paper are not restricted to the MSE but can be harnessed for the investigation of other electrodes as well. For example, the micro-channel sieve electrode (MCSE) is an RE design with extruded transit zones that form electrically isolated “micro-channels” (FitzGerald et al., 2008; Lacour et al., 2008). Recent simulations by our group have shown that the MCSE is well-suited for bidirectional interfacing of peripheral nerve tissue, as it allows for the simultaneous stimulation of sensory axons and recording of motor axons without stimulus artifact (Coker et al., 2019a,b). The combined rat sciatic nerve and behavioral model could provide an ideal platform for evaluating the MCSE’s bidirectional capabilities in an *in vivo* setting.

## Conclusion

The combined rat sciatic nerve and behavioral model is a useful tool for the characterization of an implanted electrode’s performance as a sensory feedback interface. The present study deployed this model for the measurement of detection thresholds and associated psychometric slopes for MSE multi-channel and single-channel stimulus configurations. The regenerative MSE can elicit percepts using monopolar, single-channel stimulus configurations at charge densities that range from 139 to 1,282 nC/mm^2^, which is comparable with penetrative implants. Moreover, single-channel thresholds are not uniform across the nerve, but instead are lower for the core channels and higher for the peripheral channels. Longitudinally, the observed 3-week decline for a subset of channels’ thresholds is consistent with continuing regeneration and maturation of nearby axons. These results represent an important step in establishing the MSE’s viability as a sensory feedback interface and advancing the clinical translation of this technology.

## Data Availability Statement

The raw data supporting the conclusions of this article will be made available by the authors, without undue reservation.

## Ethics Statement

The animal study was reviewed and approved by the Institutional Animal Care and Use Committee at Washington University School of Medicine in St. Louis.

## Author Contributions

NC designed the experimental apparatus, performed the main experiment and subsequent data analysis, and wrote the manuscript. NC, WM, LG, and HB developed the behavioral protocol. NC, WM, LR, ES, and NB trained the rats. NC and YY designed the head cap model and performed the surgeries jointly. HB, LG, DM, WR, and MM reviewed and edited the manuscript. All authors contributed to the article and approved the submitted version.

## Conflict of Interest

The authors declare that the research was conducted in the absence of any commercial or financial relationships that could be construed as a potential conflict of interest.

## Publisher’s Note

All claims expressed in this article are solely those of the authors and do not necessarily represent those of their affiliated organizations, or those of the publisher, the editors and the reviewers. Any product that may be evaluated in this article, or claim that may be made by its manufacturer, is not guaranteed or endorsed by the publisher.
